# The international discussion and the new regulations concerning transvaginal mesh implants in pelvic organ prolapse surgery

**DOI:** 10.1007/s00192-020-04407-0

**Published:** 2020-07-21

**Authors:** Nathalie Ng-Stollmann, Christian Fünfgeld, Boris Gabriel, Achim Niesel

**Affiliations:** 1 Department of Obstetrics and Gynecology, Medizin-Campus-Bodensee, Hospital Tettnang, Emil-Münch-Strasse 16, 88069 Tettnang, Germany; 2 Department of Obstetrics and Gynecology, St. Josefs-Hospital Wiesbaden Academic Teaching Hospital, Beethovenstr. 20, 65189 Wiesbaden, Germany; 3 Department of Obstetrics and Gynecology, University Hospital Freiburg, Hugstetter Str. 55, 79106 Freiburg, Germany; 4 Department of Obstetrics and Gynecology, Hospital Preetz, Am Krankenhaus 5, 24211 Preetz, Germany

**Keywords:** Transvaginal mesh implants, Alloplastic meshes, Pelvic organ prolapse, Urinary incontinence, Regulations

## Abstract

The use of transvaginal mesh implants for POP and urinary incontinence is currently being extensively debated among experts as well as the general public. Regulations surrounding the use of these implants differ depending on the country. Although in the USA, the UK, in Canada, Australia, New Zealand, and France, transvaginal mesh implants have been removed from the market, in most mainland European countries, Asia, and South America, they are still available as a surgical option for POP correction. The aim of this review is to provide an overview of the historical timeline and the current situation worldwide, as well as to critically discuss the implications of the latest developments in urogynecological patient care and the training of doctors.

## Introduction

Pelvic organ prolapse (POP) and urinary incontinence are stressful and quality-of-life-limiting dysfunctions. Up to 50% of women who have given birth develop POP during the course of their lives. By 2050, more than 50 million women in the USA are expected to suffer from pelvic floor dysfunctions [1]. Maintaining quality of life during old age is becoming increasingly important. Thus, there has been a rise in POP and urinary incontinence surgery in recent years.

The field of urogynecology has recently been steeped in controversy surrounding the use of transvaginal mesh implants for POP or urinary incontinence surgery. Mesh-augmented repair using polypropylene was initially introduced on the basis of the success of polypropylene mesh in the correction of incisional hernias, with the idea that POP is a type of hernia comparable with other fascial defects [2]. The successful use of polypropylene midurethral slings for urinary stress incontinence was a contributing factor to the idea of using mesh-augmented repair in POP surgery.

Not only are discussions currently being held among experts in the field, but the controversy surrounding these surgeries has also reached the media and the general public. Discussions led by patient associations, politicians, lawyers, and the press have influenced the indication for transvaginal mesh implants in surgeries for POP/urinary incontinence, resulting in different regulations being implemented in different countries [3].

In the past decade, 61 different implants for POP and urinary incontinence surgery could be temporarily found on the US market [4]. This was not only because of medical and technical progress but also because of the FDA’s Premarket Notification 510(k) approval process. According to the latter, only substantial equivalence to a precursor product was necessary for a new medical device to be approved and sold on the American market.

Worldwide, many doctors wrongly assumed that sufficient safety tests for the clinical use of transvaginal mesh implants had been carried out in advance by the FDA. This was not the case, as the “Premarket Notification” investigated only the medical device, not the surgical method. On average, 5 years passed between the approval of the first urogynecological implant and the first published clinical randomized studies [4].

The controversy surrounding transvaginal mesh implants was initially caused by a warning sent out by the FDA. The chronological order of events leading to the current worldwide situation is highlighted below.

## Timeline of the FDA safety reports

In 2007, an analysis of the Cochrane database by Maher et al. showed that better objective surgical results were obtained after transvaginal mesh repair in the anterior compartment compared with conventional anterior colporrhaphy [5]. However, there was a lack of data in regard to subjective outcome. In October 2008, the FDA released its first safety report concerning transvaginal mesh implants in response to increasing numbers of reported complications. More precise information was not available owing to a lack of published studies. In July 2011, a second safety report was published, further underlining the risk of mesh implants by concluding that adverse effects were significantly higher than expected following the use of transvaginal mesh implants and that, consequently, numerous subsequent operations had to be performed to correct these adverse effects. This review also deduced that neither the quality of life nor the effectiveness of treating POP symptoms was improved using transvaginal meshes compared with conventional techniques. It is important to note that the systematic review by the FDA used scientific literature published from 1996 to 2011. Thus, most of the transvaginal meshes investigated throughout this period are no longer available on the international market.

Owing to escalating safety concerns of the FDA, clinical studies (“Postmarket Surveillance Studies”) were imposed. Thereupon, numerous transvaginal mesh manufacturers withdrew from the American market. A survey showed that before 2011, in the USA, 90% of urogynecologists were using transvaginal mesh implants for prolapse treatment. After 2011, only 61% were still doing so [6]. This significant decline in the use of transvaginal mesh implants strongly suggests that initially these implants were implemented in a noncritical, nondifferentiated manner.

After the FDA warning in 2011, the number of lawsuits in the USA significantly increased. According to data from the Bloomberg legal database, the lawsuits were mainly directed against suburethral tapes and not against transvaginal mesh implants [7]. This is paradoxical, as to date, suburethral slings have been firmly established as the gold standard in the treatment of female stress urinary incontinence. Moreover, implant manufacturers were sued in 99.4% of the cases, while treating physicians were sued in only 0.6% of all cases [7]. Of these physicians, only 12% were certified for POP and urinary incontinence surgeries by their respective medical boards [7].

In January 2016, the FDA decided to further increase the risk class of transvaginal meshes from risk class II to class III. This meant that extensive clinical studies (“premarket approvals”), the agency’s most strict device review pathways, were now required for all transvaginal mesh implants. The FDA decreed that all premarket approval applications had to be filed by 5 July 2018. Consequently, all manufacturers stopped marketing transvaginal meshes intended for posterior repair in the USA. Only two companies decided to conduct studies on anterior transvaginal mesh implants over a period of 3 years. Interestingly, suburethral slings and mesh implants for abdominal repair remained in risk class II and required no premarket approvals.

In April 2019, despite lacking the results of the 36-month follow-up data, which had been demanded by the FDA in 2016, the FDA surprisingly decided to promptly disallow the sales of anterior transvaginal mesh implants. The reason given was the apparent lack of proof of superiority of transvaginal anterior mesh repair over conventional anterior colporrhaphy.

The FDA’s decision caused a worldwide stir among urogynecological experts. Specialists supporting the FDA’s decision were opposed to a petition started and supported by other specialists, which collected over 10,000 signatures and urged for the continued use of transvaginal meshes in POP surgery, albeit in a targeted and critical manner [8].

## Situation in the UK, Australia, New Zealand, and Canada

In the UK, the discussion was influenced by the PROSPECT study [9]. In this study, patients with primary POP were randomized into three groups: conventional anterior colporrhaphy, anterior mesh repair with synthetic implants, and anterior mesh repair with biological implants. They were subsequently compared in a controlled manner. No differences in prolapse-related quality of life and side effects (infection, dyspareunia, bladder-emptying disorder) were found. However, 12% of all complications were mesh-related [9]. The study stood in contrast with the systematic review of the Cochrane database of 2016, which had shown fewer anatomical recurrences and less subjective POP upon the use of transvaginal mesh implants compared with conventional colporrhaphy [9, 10].

The PROSPECT’s study design was pragmatic and aimed at high evidence under everyday conditions. To avoid bias, a high number of patients was recruited as well as a high number of surgeons. Each surgeon operated with his/her preferred meshes and surgical techniques, i.e., above or below the transvaginal fascia. In the two mesh-based study arms, the nowadays obsolete inlay technique was used. Apical fixation, which is considered essential in achieving successful POP repair in the anterior compartment by some authors, was not discussed [11]. Only the mesh type was standardized, i.e., Amid type 1. It was permitted to insert light and heavy meshes. The extent to which surgical expertise plays a role in the study outcome is debatable, as 52% of the surgeons had inserted fewer than 10–20 meshes (transvaginal mesh kits) before the start of the study [12]. Futyma et al. criticize the low average number of patients per center, as well as the incongruent assignment of patients into subgroups [13]. A total of 1,348 patients, with 803 patients receiving transvaginal mesh implants, were operated on in 35 centers. Given the recruitment period of 3 years and 8 months, this translates to an average of only 0.5 transvaginal mesh implant procedures per month, per center. Last, the authors of the PROSPECT study also stated that a better outcome can probably be achieved through the selection of suitable patients, standardized surgical technique, and specially trained surgeons [9].

In 2017, the NICE (National Institute for Care and Health Excellence) draft guidance recommended that mesh-augmented POP repair should only be used in clinical studies. In July 2018, Baroness Cumberlege, chair of the Independent Medicines and Medical Devices Safety Review, called for a halt in the use of transvaginal meshes until conditions affecting the training of surgeons, their complications, as well as their licensing, were met. The only possibility of using the latter was when no alternative options were available.

In June 2019, the NICE updated its stand on mesh implants with retropubic slings (in a bottom-up technique) once again being offered as a treatment option alongside the Burch colposuspension, the autologous rectus fascia sling, and bulking agents for urinary incontinence surgery. In contrast, TOT slings (exception: retropubic adhesions), retropubic slings inserted in a top-down technique, and artificial sphincters are still prohibited. In the update, transvaginal meshes in the anterior compartment were only to be implanted under study conditions and the posterior compartment was to be corrected without mesh, using conventional techniques. Abdominal or laparoscopic sacrocolpopexy and sacrohysteropexy using abdominal meshes could still be performed. According to a recent statement by the House of Commons, the use of transvaginal meshes is still on hold. Similar decisions have been taken in Scotland, Wales, and Ireland.

In Australia, transvaginal meshes are currently not available on the market. However, suburethral slings for urinary incontinence are still considered safe to use. In New Zealand, suburethral slings can still be implanted, but not transvaginal meshes.

In July 2019, Health Canada issued a safety review in which transvaginal meshes for posterior repair were to be removed from the Canadian market [14]. According to the review, transvaginal meshes for anterior and apical repair are only to be used in patients at a high risk for recurrence of POP, or for whom alternative treatment options are not suitable [14]. Subsequently, the three companies with active licenses in Canada in 2019 withdrew their products from the market [14].

## Situation in Asia

The training and practice of urogynecology in Asia is very diverse. Augmented synthetic self-cut mesh POP surgery is more commonly performed in Japan, where ready-made synthetic mesh kits are not licensed by the national medical regulatory authority [15]. Ready-made mesh kits are mainly used by urologists in South Korea, as well as by some urogynecologists in Taiwan, Hong Kong, Singapore, Thailand, Malaysia, Indonesia, and India [15]. In China, both self-cut meshes as well as ready-made mesh kits are used [15].

## Situation in mainland Europe and South America

In South America, there are currently no prohibitions concerning transvaginal mesh implants. In France and the Netherlands, limitations have been imposed. Although in the Netherlands transvaginal meshes are approved in certified facilities during regular audits, in France, the use of transvaginal mesh implants has recently been prohibited, except in the context of clinical studies.

The EU’s position in Brussels has remained unchanged since the Scientific Committee on Emerging and Newly Identified Health Risks (SCENIHR) report of 2015, i.e., vaginal meshes can be used in the case of POP recurrence or in cases in which the patient is at a high risk for recurrence, a certification system for surgeons should be introduced, and patients should be appropriately selected and counseled [16]. The essentially concurring positions of 24 different medical societies on the use of transvaginal implants were published in a FIGO Review of 2019, which stated that these should only be used for complex indications, that the “informed consent” of the patient is required, and that each surgeon’s own data have to be collected and clinical studies have to be performed [3].

## Situation in Germany, Austria, and Switzerland

In addition to the SCENIHR recommendations, the current guideline of the German Working Group of the Scientific Medical Societies (AWMF), the European Urogynecological Association (EUGA) and the European Association of Urology (EAU) permit the differentiated use of synthetic transvaginal meshes in POP repair under certain conditions [17]. In contrast to the FDA, in the German–Austrian–Swiss guideline it was deduced that transvaginal mesh implants are superior in terms of objective success rates and recurrence rates compared with conventional POP surgery; native tissue repair with apical fixation had a cumulative success rate of 69%, whereas anterior mesh repair with apical fixation had a cumulative success rate of 93% [17]. A metanalysis in the German–Austrian–Swiss guideline showed that the POP recurrence rate was three times higher in native tissue repair than in anterior mesh repair [17]. Anterior transvaginal mesh implants should primarily be used in patients with recurrence or in a primary POP situation, when a high risk of recurrence is expected, in cases of avulsion of the levator ani muscle, when the patient has a great need for anatomical stability owing to, for example, a physically strenuous job, the patient has high levels of comorbidity making subsequent operations upon POP recurrence risky for the patient, or the patient has specific risk factors for POP recurrence, such as chronic asthma or COPD [17]. Crucially, the patient has to be informed about all treatment options in the sense of “shared decision making”, i.e., observation, conservative therapies (physiotherapy, pessaries), and different surgical treatment options. Evidence also suggests that patient-centered goal achievement, in the sense of symptom relief and improved activity following pelvic floor surgery, can be associated with improved quality of life and overall satisfaction [18]. In Germany, during the past 15 years, there have been continued development and improvement of transvaginal meshes [19–21]. Meshes from the FDA reports are no longer available on the German market and have been replaced with lighter, macroporous, isoelastic meshes that have significantly lower mesh erosion rates.

The German Association of Urogynecologists (AGUB) has introduced a level I to III certification of physicians according to their expertise in urogynecology. Level III identifies surgeons qualified for complex POP operations, involving mesh implants. The AGUB promotes the establishment of specialized centers. In 2019, a unified certification for continence and pelvic floor centers in Germany under the umbrella of the German Society for Gynecology and Obstetrics (DGGG), German Society for Urology (DGU), German Society for General and Visceral Surgery (DGAV), German Hospital Federation (DKG), and the German Continence Society was established. By forming these centers, the use of transvaginal mesh implants in accordance with the guidelines is guaranteed. A complications register, in which all surgeons are requested to enter their data, has been set up by the AGUB. A German general implant register, which also includes urogynecological medical devices, is currently being planned.

## Outlook

It is obvious that in the USA, in the UK, in Australia, New Zealand, Canada, and France, with the removal or restriction of transvaginal mesh implants as a surgical option for POP, the spectrum of urogynecological operations will be greatly affected. Conventional transvaginal native tissue repair and abdominal (open, laparoscopic) surgical procedures are expected to be increasingly performed. This requires appropriate training of younger physicians. In Scotland, owing to the FDA notification, incontinence surgery was reduced by 86% [22]. This decrease mainly affected suburethral slings. Surprisingly, the number of conventional surgeries for urinary incontinence, e.g., Burch colposuspension, did not increase in a compensatory manner. Simultaneously, the number of POP surgeries decreased by 37%, mainly because of less frequent colporrhaphies [22]. Laparoscopic sacrocolpopexy and sacrohysteropexy were performed so rarely that they were not included in a retrospective study by Morling et al. [22].

Mesh-related complications are not only due to surgical training or the mesh material. Recently, it has been discussed that mesh erosions may be related to infections and a disturbed vaginal microbiome [23]. Groin pain after extensive prolonged reconstructive surgery may be due to microtears in the ligaments, tendons, and muscles located in the groin and not due to the mesh itself. According to Jeffery and Roovers, there is a demand for research into new mesh material, i.e., textile and type of biomaterial to improve the biochemical characteristics and immune resistance [24]. Clinical research should focus on long-term outcomes, ideally 10–15 years for objective and subjective results. Subjective outcomes could measure quality of life in different aspects, such as POP symptoms, sexual activity, bowel symptoms, and urinary tract symptoms. Objective outcomes, using POP-Q measurements, should be standardized to achieve better consensus. Surgical methods should also be optimized and standardized. Moreover, international standardized complication registers should be established. Complication registers are currently being handled differently in different countries, which makes data collection difficult. In Sweden, a nationwide register with patient-reported function and outcome data is available; in the USA, patients and doctors report complications to the FDA; in the UK, a mandatory complications register for surgeons, imposed by the government, is being set up, and the complications register of the IUGA is voluntary for its members.

## Conclusion

An increasing number of women are suffering from POP and urinary incontinence worldwide. At the same time, there is an increasing wish to maintain quality of life in old age. A wide range of reconstructive surgical procedures is offered in operative urogynecology. Transvaginal mesh implants for the treatment of POP are being extensively debated as a result of complications. Following the FDA marketing stop in the USA, their use in the UK, Canada, Australia, New Zealand, and France was also discontinued or paused. These decisions have, however, been driven by controversially led discussions and political interests, not necessarily by scientifically led facts.

According to the SCENIHR recommendation and the joint German–Austrian–Swiss guideline, transvaginal mesh implants should be used in the event of recurrence and may, in certain cases, even be a surgical option in a primary situation. It is crucial that patients be treated individually and that the advantages and disadvantages of meshes be considered with regard to individual findings. Clear indications and surgical expertise, including extensive training of surgeons, complication management in specialized urogynecological centers, monitoring of complications, as well as the ongoing improvement of transvaginal meshes, are critical for transvaginal mesh implants to continue to be an option in POP surgery.
